# *PiggyBac*-Generated CAR19-T Cells Plus Lenalidomide Cause Durable Complete Remission of Triple-Hit Refractory/Relapsed DLBCL: A Case Report

**DOI:** 10.3389/fimmu.2021.599493

**Published:** 2021-05-25

**Authors:** Chenggong Li, Yan Sun, Jing Wang, Lu Tang, Huiwen Jiang, Tao Guo, Lin Liu, Yaohui Wu, Lisha Ai, Linghui Xia, Jianjun Wu, Zhicai Lin, Qijun Qian, Yu Hu, Heng Mei

**Affiliations:** ^1^ Institute of Hematology, Union Hospital, Tongji Medical College, Huazhong University of Science and Technology, Wuhan, China; ^2^ Hubei Clinical Medical Center of Cell Therapy for Neoplastic Disease, Wuhan, China; ^3^ Shanghai Cell Therapy Group Co. Ltd., Shanghai, China; ^4^ Radiology Department, Union Hospital, Tongji Medical College, Huazhong University of Science and Technology, Wuhan, China

**Keywords:** triple-hit lymphoma, diffuse large B cell lymphoma, relapsed/refractory, chimeric antigen receptor-T cell therapy, *PiggyBac* transposon system, lenalidomide maintenance

## Abstract

MYC/BCL2/BCL6 triple-hit lymphoma (THL) is an uncommon subset of high-grade B-cell lymphoma with aggressive clinical behavior and poor prognosis. TP53 mutation is an independently poor progonistic indicator in patients with THL, hence novel therapeutic strategies are needed for these patients. CD19-directed chimeric antigen receptor(CAR19)-T cell therapy has shown promising efficacy for relapsed/refractory diffuse large B cell lymphoma (RR DLBCL), but the majority of CAR19-T cell products to date have been manufactured using viral vectors. *PiggyBac* transposon system, with an inclination to memory T cells, offers a more convenient and economical alternative for transgene delivery. We herein report the first case of triple-hit RR DLBCL with TP53 mutation who was treated with *piggyBac-*generated CAR19-T cells and accompanied by grade 2 cytokine release syndrome. The patient obtained a complete remission (CR) in the 2nd month post-infusion and demanded maintenance therapy. Whether maintenance therapy is favorable and how to administrate it after CAR-T cell infusion remain controversial. Preclinical studies demonstrated that lenalidomide could enhance antitumor activity of CAR19-T cells. Therefore, we pioneered oral lenalidomide after CAR19-T therapy in the patient from the 4th month, and he discontinued after one cycle due to side effects. The patient has still kept sustained CR for over 24 months. Our case have firstly demonstrated the feasibility, preliminary safety and efficacy of *piggyBac*-produced CAR19-T cell therapy in triple-hit lymphoma. The innovative combination with lenalidomide warrants further investigation. Our findings shed new light on the possible solutions to improve short-term relapse after CAR19-T cell therapy in RR DLBCL. ChiCTR, number ChiCTR1800018111.

## Introduction

Diffuse large B cell lymphoma (DLBCL) is the most common subtype of aggressive non-Hodgkin lymphoma, accounting for 30–40% of newly diagnosed cases worldwide (1). Although most patients achieve remission with R-CHOP (rituximab, cyclophosphamide, adriamycin and prednisone) immunochemotherapy, 10–15% exhibit primary refractory disease and 20–35% suffer a relapse (2). For those with relapsed/refractory (RR) DLBCL, the median overall survival (OS) was only 6.3 months with conventional therapy (3). Triple-hit lymphoma (THL) that carries concurrent MYC, BCL2 and BCL6 rearrangements is a relatively rare subset, identified in approximately 1% of DLBCL patients (4–6). These rearrangements result in highly aggressive clinical behavior, resistance to standard chemotherapy and extremely poor outcomes (7). Although high-dose chemotherapy combined with autologous stem cell transplantation (ASCT) remains the standard treatment for RR DLBCL, a series of studies demonstrated no appreciable benefits or even inferior outcomes for patients with THL after ASCT (8–10). TP53 is an important tumor suppressor gene and is proven as an inferior prognostic factor in DLBCL (11). Novel therapeutic strategies are needed to improve survival for these patients with triple-hit RR DLBCL.

CD19-specific chimeric antigen receptor (CAR19)-T cell therapy has offered a new paradigm for the treatment of RR DLBCL. Three second-generation CAR19-T cell products, axicabtagene ciloleucel (axi-cel), tisagenlecleucel (tisa-cel) and lisocabtagene maraleucel (liso-cel) have got FDA-approval for DLBCL (12–14). A latest meta-analysis demonstrated that second-generation CAR19-T cell therapy attained a remarkable overall response rate (ORR 55–79%) in RR DLBCL (n = 306), and the median OS was 13.2 months (15). Subgroup analyses demonstrated that CAR19-T cell therapy exhibited consistent efficacy between doublt-hit or triple-hit lymphoma and standard-risk DLBCL, supporting its application in triple-hit RR DLBCL (16, 17). The majority of CAR-T cells used in clinical trials to data are conducted by lentivirus or retrovirus. *PiggyBac* transposon system, as an emerging non-viral methodology for stable genetic modification of human T cells, possesses a large gene-capacity, simple and cost-effective manufacturing, and an inclination to stem-cell memory (SCM) phenotype (18–21). Preclinical studies suggested that *PiggyBac-*generated CAR19-T cells had a potent activity against B-cell malignancies (22–24). However, the efficacy and safety of *piggyBac*-engineered CAR-T cells haven’t been reported in human clinical trials. Here, we report the first case with triple-hit RR DLBCL who has received *piggyBac*-generated CAR19-T cell therapy and achieved durable complete remission (CR) for over 24 months.

Whether maintenance treatment is favorable and how to administrate it after CAR19-T cell therapy are hotly debated issues in RR DLBCL. Preclinical studies demonstrated that lenalidomide could enhance antitumor function of CAR19-T cells for lymphoma (25). Lenalidomide has been approved as maintenance therapy after transplantation for multiple myeloma, but not reported as maintenance after CAR-T cell therapy. The patient obtained a CR in the 2nd month and demanded maintenance therapy. Therefore, we have pioneered oral lenalidomide in the patient after CAR19-T cell infusion, which is worth further exploration.

## Case Report

### Lymphoma Treatment History

**Figure 1:**
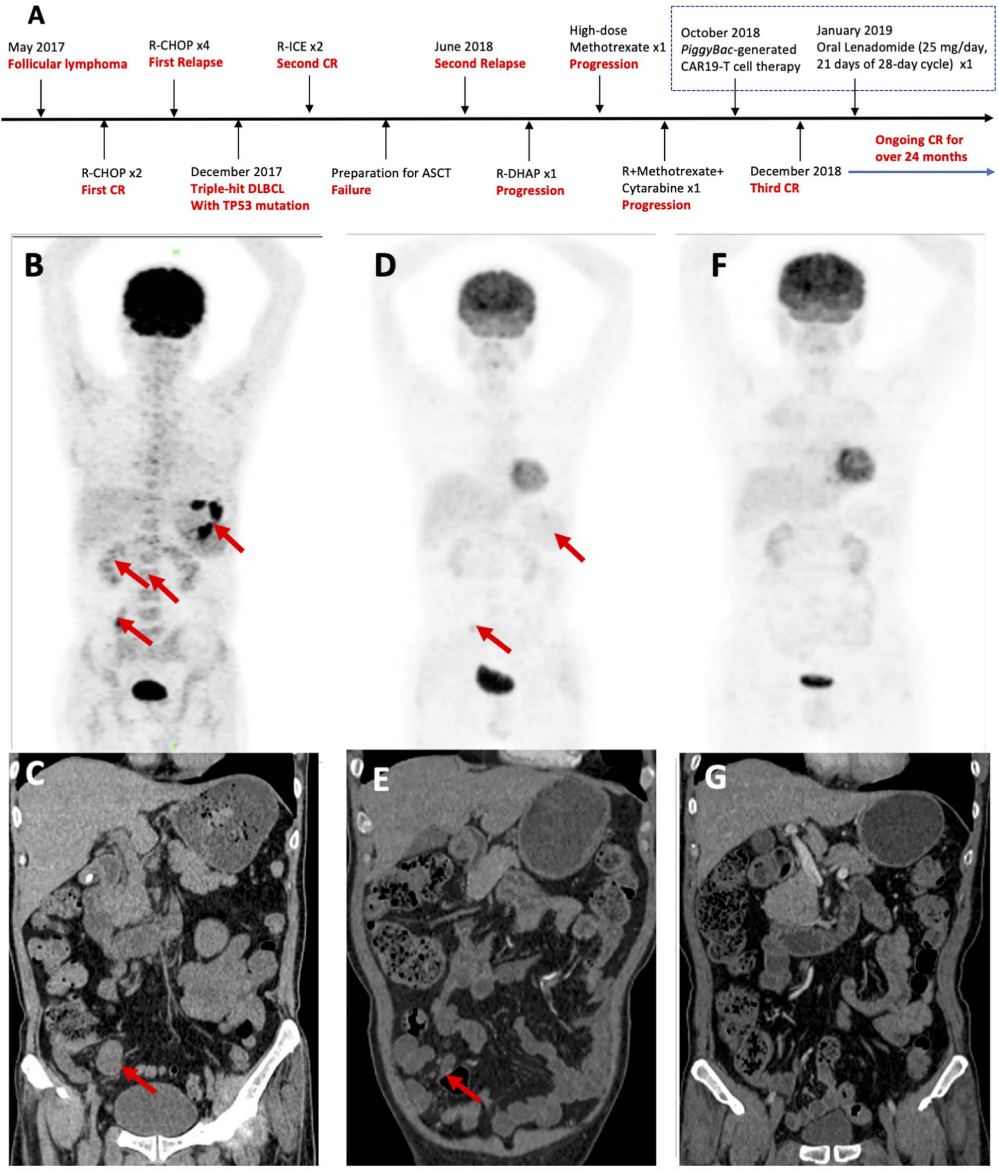
Patient treatment history and response to *piggyBac*-generated CAR19-T cells. **(A)** Patient’s disease progression and lymphoma treatment history. **(B)** Pre-treatment PET-CT, showing extensive invasion of tumors in gastric wall (SUVmax 13.2–18.0), small intestines (SUVmax 6.7) and bone marrow (SUVmax 2.6–3.2), scoring 5 points per Deauville criteria. **(C)** Pre-treatment abdominal CT, showing an abnormal mass sized as 3.0 × 2.2 cm (arrow). **(D)** PET-CT in the 1st month post-infusion, showing diminished invasion of tumors in small intestines (SUVmax 4.6) scoring 3 points per Deauville criteria. **(E)** Abdominal CT scans in the 1st month, showing an abnormal mass sized as 1.6 × 1.0 cm (arrow). **(F)** PET-CT scans were consistent with a complete metabolic response in the 2nd month post-infusion. **(G)** Normal Abdominal CT in the 2nd month.

A 53-year-old Chinese male patient was diagnosed as follicular lymphoma (grade 3A, stage II, group B) in May 2017 and achieved first CR after two cycles of R-CHOP (**Figure 1**). After given four cycles of R-CHOP, the patient’s disease relapsed. The pathologic biopsy and immunohistochemistry (IHC) of celiac lymph nodes revealed DLBCL, activated B-cell (ABC) subtype, and overexpression of MYC, BCL2 and BCL6. Fluorescence *in situ* hybridization (FISH) of the lymphoma tissues also detected the triple rearrangements. Second-generation sequencing of the paraffin-embeded lymphoma tissues indicated 68.70% of TP53 mutation. The patient receieved two cycles of R-ICE (rituximab, ifosfamide, carboplatine and etoposide), and obtained a second CR. Unfortunately, he failed to collect hematopoietic stem cells for ASCT, and his disease progressed again with bone marrow involvement in June 2018. He complained of pain and weakness of both lower limbs, especially the left lower extremity. Subsequently, one cycle of R-DHAP(rituximab, dexamethasone, cytarabine and cisplatine) was administrated. Symptoms of left peripheral facial paralysis and severe headache occurred, and supportive treatment showed no response. The patient then suffered left eyeball pain, but still kept complete self-cognition. Head computed tomography (CT) scans excluded elevated intraocular pressure and intracranial space-occupying lesions. Head magnetic resonance imaging, including plain and enhancement scans, didn’t show involvement of the brain parenchyma. The patient was unable to receive lumbar puncture and intrathecal chemotherapy because of pain and weakness of both lower limbs. He was given promptly with high-dose methotrexate intravenously once and single infusion of combined regimens including rituximab, high-dose methotrexate and cytarabine. Positron emission tomography (PET)-CT scans suggested extensive invasion of lymphoma in gastric wall, small intestines and bone marrow, scoring 5 points per Deauville criteria (**Figure 1**). Abdominal CT scans showed an abnormal mass sized as 3.0 × 2.2 cm (**Figure 1**). Repeated IHC of the paraffin-embeded lymphoma tissues was strongly positive for CD19. 1.5% of the cells were of unknown classification in bone marrow cytology, but no abnormal monoclonal B cells were found in bone marrow immunophenotyping. FISH showed the triple rearrangements of MYC, BCL2 and BCL6, and 4.51% of TP53 mutation was found in bone marrow. Cerebrospinal fluid examination didn’t indicate involvement in central nervous system. Therefore, the patient was enrolled in the phase 1 study (ChiCTR1800018111).

### Manufacturing of *piggyBac-*Generated CAR19-T Cells

**Figure 2:**
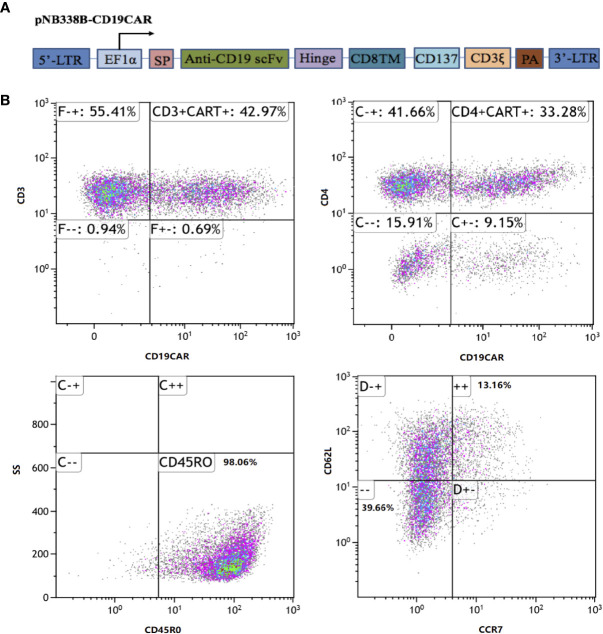
CD19CAR structure and immunophenotype of infused *piggyBac*-generated CAR19-T cell products. **(A)** Schematic diagram of CD19CAR. SP, CDS signal peptide; TM, Transmembrane; PA, SV40 Poly A signal. **(B)** In the final infused products, the CAR transfection efficiency was 42.97%, including 33.28% of CD4^+^CAR^+^ cells and 13.54% of stem-cell memory T cells (CD45RO^+^CCR7^+^CD62L^+^).

The CD19CAR incorporated an FMC63 mAb-derived single chain variable fragment, a human CD8α hinge and transmembrane domain, an intracellular 4-1BB (CD137) costimulatory domain, and a cytoplasmic CD3ζ signal (**Figure 2**). The CD19CAR gene was cloned into the *PiggyBac* transposon vector pNB328-EF1α to construct pNB328-CD19CAR, as described (26). Peripheral blood mononuclear cells were collected by leukapheresis from the patient and isolated by Ficoll density gradient centrifugation. T cells were electroporated with pNB328-CD19CAR plasmids and then activated by anti-CD3/CD28 antibodies in KBM581 medium containing 200 U/ml recombinant human interleukin (IL)-2 for 5 days. Thereafter, the activated cells were cultured until meeting the predefined release criteria, including transduction efficiency ≥5%, cell viability ≥70%, negative mycoplasma, negative bacterial and fungal cultures.

### CAR19-T Cell Infusion and Patient’s Outcomes

The patient was given the lymphodepleting chemotherapy(fludarabine 50mg, days-5 to -3; cyclophosphamide 1,200 mg, day-3). On October 23, 2018 (day 0), the patient received an infusion of *piggyBac*-generated CAR19-T cells at the dose of 1.0 × 10^6^/kg. In the final infused cell products, the CAR transfection efficiency was 42.97%, including 33.28% of CD4^+^CAR^+^ cells and 13.54% of SCM T cells (**Figure 2**).

**Figure 3:**
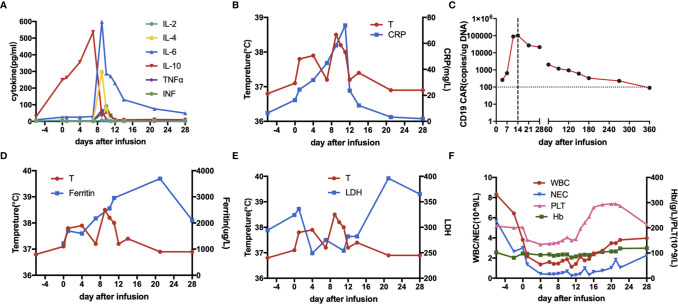
Clinical monitoring after infusion of piggyBac-generated CAR19-T cells. **(A)** Trends of Interleukin(IL)-2, IL-4, IL-6, IL-10, tumor necrosis factor α (TNFα) and interferon (INF) during 28 days after infusion. **(B)** Trend of C-reactive protein (CRP) was associated with temperature (T) rising during 28 days after infusion. **(C)** CAR19 copies in peripheral blood were measured by quantitative polymerase chain reaction. **(D, E)** The peak values of ferritin and lactate dehydrogenase (LHD) after infusion were later than temperature. **(F)** Changes of white blood cells (WBC), neutrophils (NEC), platelet (PLT) and hemoglobin (Hb) during 28 days after infusion.

During days 9 to 11 post-infusion, the patient experienced pyrexia, hypoxia and emesis with a maximum temperature of 39.3 °C, which was rated as grade 2 cytokine release syndrome (CRS) against Lee’s criteria (27). As the body temperature rose after infusion, serum IL-6 and C-reactive protein significantly elevated, peaked at 598.5 and 73.8 mg/l, respectively (36-fold and 9-fold over upper limit of normal) (**Figure 3A**). Therefore, intravenous tocilizumab 8 mg/kg and supportive treatment were administrated, and CRS got well controlled. The CAR copies increased dramatically and peaked at 103,000 copies/ug on day 14 (**Figure 3**). The peak of ferritin and lactate dehydrogenase were detected on day 24, later than the time when toxicity culminated (**Figure 3D**). Moreover, he developed grade 3–4 hematological toxicities, which were relieved during the 1st month (**Figure 3**).

The patient obtained a partial remission (PR) in the 1st month per Lugano criteria (**Figure 1D**) (28). Bone marrow biopsy and immunophenotyping had no signs of lymphoma involvement, and only 0.5% of the cells of unknown classification were found in bone marrow cytology. In the 2nd month, PET-CT scans were consistent with a complete metabolic response, and abdominal CT scans were normal (**Figure 1F**). Bone marrow examination were normal, including biopsy, cytology, and immunophenotyping. TP53 mutation disappeared and FISH detected no abnormality in bone marrow. In the 3rd month, the patient maintained CR and demanded maintenance therapy. Oral lenadomide(25 mg/day, 21 days of 28-day cycle) was given in the 4th month and the patient discontinued after one-cycle administration due to side effects such as skin rashes, pruritus and painful joints. The CAR copies could be detected in peripheral blood until the 9th month post-infusion by quantitative polymerase chain reaction (**Figure 3**). The patient has still kept durable CR for over 24 months.

## Discussion

To the best of our knowledge, this is the first case with triple-hit RR DLBCL who has received *piggyBac*-generated CAR19-T cell therapy and maintained durable CR for over 24 months. It’s also the first report about oral lenalidomide maintenance after CAR-T cell infusion.

The 2016 revised World Health Organization guidelines of lymphoid neoplasms classified large B-cell lymphoma with rearrangements of MYC and BCL2 or/and BCL6 in a distinct category to be designated high-grade B-cell lymphoma, also called double-hit lymphoma (DHL) or THL (4). DHL comprises approximately 2–10% of newly diagnosed DLBCL cases, and THL is a rare subset, accounting for almost 1% in DLBCL (5, 6). The largest series of THL to data included 40 patients, suggesting that its clinicopathologic features were similar to DHL and TP53 mutation was an independent predictor of poor prognosis (7). Patients with DHL/THL have significantly suboptimal responses and dismal outcomes with standard first-line R-CHOP chemoimmunotherapy (6, 29, 30). Our case relapsed following initial CR to R-CHOP and transformed to triple-hit DLBCL. High-dose chemotherapy combined with ASCT has historically regarded as the curative chance for patients with chemotherapy-sensitive relapse (31). Therefore, our patient was given R-ICE as salvage regimens prior to ASCT, and the patient achieved second CR. Unfortunately, the patient failed in the bridge to ASCT, and suffered second recurrence, and even became resistant to another three lines of salvage chemotherapy. Novel therapeutic options are urgently needed for these patients with triple-hit RR DLBCL.

CAR19-T cell therapy has emerged as a novel promising immunotherapy exhibiting remarkable efficacy in patients with chemotherapy-refractory DLBCL (**Table 1**). Axi-cel, as the first FDA-approved CAR19-T cell product for RR DLBCL, is generated untilizing retroviral vectors and contains a CD28 costimulation domain (12). In the pivotal ZUMA-1 trial, axi-cel showed a striking ORR and CR rate of 83 and 58% (n = 108), respectively. At a median follow-up of 27.1 months, 39% maintained ongoing remission, and the median OS didn’t reach. 93% of patients had CRS per Lee criteria (severe 11%), and 67% experienced neurotoxicity (severe 32%), both of which were manageable and largely reversible (32). Tisa-cel is the second FDA-approved CAR19-T cell product for DLBCL, which is manufactured using lentiviral vectors and a 4-1BB costimulation domain (13). In the pivotal JULIET study, 52% of patients achieved an objective response and 40% attained a CR (n = 93) at a median follow-up of 14 months. CRS occurred in 64% of patients (severe 22%) against the Penn grading scale, and neurotoxicity occurred in 23% of patients (severe 12%) (16). The lower incidence of CRS and neurotoxicity compared with axi-cel is possiblely related to 4-1BB costimulation domain ultilized in tisa-cel. Parallel comparison of 4-1BB or CD28 co-stimulated CAR19-T cells for B-cell lymphoma suggests that 4-1BB is more beneficial and tolerated for the clinical performance (33). Therefore, 4-1BB is widely applied in subsequent CAR-T cell products, including lisocabtagene maraleucel(liso-mar) and the *piggyBac*-generated CAR19-T cells in our study. Liso-mar is being actively tested in B-cell lymphoma. In the TRANSCEND trial, liso-mar exhibited a high response rate (73%, n = 268) and low incidences of CRS (42%, severe 2%) and neurotoxicity (30%, severe 10%) (14). Subgroup analyses of the JULIET and TRANSCEND trials demonstrated that patients with DHL/THL responded as similarly well to CAR19-T cell therapy as standard-risk DLBCL (14, 16, 17). These results support the application of CAR19-T cell therapy in doublt-hit or triple-hit RR DLBCL.

**Table 1:** Representative CAR19-T cell products in B-cell lymphoma.

CAR19-T Cell Products	Axicabtagene Ciloleucel[11]	Tisagenlecleucel[12]	Lisocabtagene Maraleucel[13]
CAR Construct	FMC63(CD19 scFv)/CD28/CD3ζ	FMC63(CD19 scFv)/4-1BB/CD3ζ	FMC63(CD19 scFv)/4-1BB/CD3ζ CD4:CD8=1:1
Vector	Retrovirus	Lentivirus	Lentivirus
FDA-approved indication	RR DLBCL, PMBCL, transformed FL, and HGBCL	RR DLBCL, PMBCL, transformed FL, and HGBCL	RR DLBCL, HGBCL, PMBCL, and FL grade 3B
Clinical Trial	ZUMA-1(NCT02348216)	JULIET(NCT02445248)	TRANSCEND(NCT02631044)
CAR-T Cell Dose	2×10^6^ cells/kg	median 3×10^8^ cells/kg range (0.1-6.0) ×10^8^ cells/kg	DL1 5×10^7^ total cells[once/twice] DL2 10×10^7^ total cells DL3 15×10^7^ total cells
Enrolled Patients	119, 108 infused	165, 111 infused	344, 269 infused
Bridging Therapy	not allowed	92%	59%
Lymphodepletion Regimens	Cy 500mg/m^2^+Flu 30mg/m^2^ on day-5, -4 and -3	Cy 250mg/m^2^ +Flu 25mg/m^2^ for 3 days or Ben 90mg/m^2^ for 2 days	Cy 300 mg/m^2^ + Flu 30mg/m2 on day-5, -4 and -3
Efficacy Evaluation	n=101	n=93	n=256
Best ORR	83%	52%	73%
Best CR rate	58%	40%	53%
Median Follow-up	27.1m (IQR 25.7-28.8)	14m (range 0.1-26)	Not reported
Median DOR	11.1m (range 4.2- NR)	NR (95%CI 10-NR)	NR (95%CI 8.6- NR)
Median PFS	5.9m (95%CI 3.3-15.0)	12m-PFS 83%	6.8m (95%CI 3.3-14.1)
Median OS	NR (range 12.8- NR)	12m (95%CI 7.0- NR)	21.1m (range 13.3- NR)
Relapse Rate	53.5% (45/84)	65% responders remain relapse-free at 12m	Not reported
Safety Evaluation	n=108	n=111	n=268
CRS	93%(severe 11%)	64%(severe 22%)	42%(severe 2%)
Neurotoxicity	67%(severe 32%)	23%(severe 12%)	30%(severe 10%)

PMBCL, primary mediastinal B-cell lymphoma; FL, follicular lymphoma; HGBCL, high-grade B-cell lymphoma; Flu, fludarabine; Cy, cyclophosphamide; Ben, bendamustine; DOR, duration of remission;PFS, progression-free survival; m, month(s); IQR, interquartile range; CI, confidence interval; NR, Not Reached.

The majority of CAR-T cells used in clinical trials to data are conducted by lentivirus or retrovirus. Paralleled comparison of vectors for the generation of CAR-T cells was comprehensively reviewed (34). Viral vectors possess an ideal transduction efficiency and stable transgene expression, but to some extent, they also correlate with a high risk of insertional mutagenesis and potential malignant transformation. The novel non-viral *PiggyBac* transposon system, with decreased integration frequency into proto-oncogenes in human T cells, shows great application potential for stable genetic modification of human T cells (35). *PiggyBac* system consists of two components: a transposon plasmid carrying the target gene and another plasmid encoding the transposase, both of which are introduced into cells by electroporation. Preclinical studies suggested that *PiggyBac-*generated CAR19-T cells had a potent activity against B-cell malignancies (22–24). However, the preliminary efficacy and safety of *piggyBac*-engineered CAR-T cells haven’t been reported in human clinical trials. Here, we report the first case with triple-hit RR DLBCL who has received *piggyBac*-generated CAR19-T cell therapy. Limited *in vivo* expansion and persistence of CAR19-T cells is considered as a main possible mechanism of CD19-positive relapse after CAR19-T cell therapy (36). SCM T cells are known to promote superior *in vivo* perliferation and survival of CAR-T cells (37–40). Previous studies indicated that *piggyBac* system had the preference to SCM T cells (21, 23, 41), thus *piggyBac* system could be a feasible optimization for CAR-T manufacturing. In the case, the final infused cell products contained only 13.54% of SCM T cells and owned *in vivo* lifespan of 9 months, which inadequately explained the patient’s exceptional response.

Whether maintenance treatment is favorable and how to administrate it after CAR19-T cell therapy are hotly debated issues in RR DLBCL. The patient obtained a CR in the 2nd month and demanded maintenance therapy. Despite ASCT recommended as the standard care for RR DLBCL, a series of studies demonstrated no appreciable benefits or even inferior outcomes for patients with DHL/THL after ASCT (8–10). Until now there is no evidence to support consolidation with transplantation after CAR-T cell therapy in lymphoma, hence novel strategies for maintenance therapy are warranted to explore. Lenalidomide, an oral immunomodulator, has been approved by FDA as maintenance therapy after ASCT for patients with multiple myeloma based on evidences from two randomized, blinded trials (CALGB100104 and IFM 2005-02) (42–44). Lenalidomide also exhibited activity as maintenance therapy in RR DLBCL, especially in ABC subtype for the reason that lenalidomide strongly inhibited NF-kB signaling, key pathogenesis of ABC subtype (45). Oral lenalidomide maintenance (25 mg/day, 21 days of 28-day cycle) after salvage chemotherapy in patients with RR DLBCL (n = 47) attained 1-year and 5-year PFS of 70 and 53%, respectively (46). Preclinical studies demonstrated that lenalidomide could enhance antitumor function of CAR19-T cells for aggressive B-cell lymphoma, whose mechanisms included augmented cytotoxicity, memory maintenance and persistence, and Th1 cytokine production (25). These results indicated that lenalidomide maintenance after CAR-T cells therapy deserved investigation. Therefore, we pioneered oral lenalidomide in the patient in the 4th month. The patient still maintains CR for over 2 years and his OS is over 3 years.

Several limitations exist in the present study. First, this is a case report, and more cases are needed to observe the overall safety and efficacy of *piggyBac*-generated CAR19-T cell therapy in RR DLBCL. Second, the patient discontinued after one-cycle lenalidomide due to adverse reactions, thus the risks of lenolidomide shouldn’t be understated. A multicenter retrospective study indicated that lymphoma patients had similar benefits in ORR, PFS and OS when administrated with 10, 15 or 25 mg/day lenalidomide (47). Oral low-dose lenalidomide maintenance (10 mg) after CAR-T cell therapy deserves further exploration to minimize side reactions. Third, the additive role of lenalidomide maintenance after CAR-T cell therapy warrants controlled trials to verify.

In conclusion, our case, for the first time, has demonstrated the feasibility, preliminary safety and efficacy of *piggyBac*-produced CAR19-T cell therapy in triple-hit lymphoma. Future investigations with large sample sizes are needed to clarify the overall safety and activity of *piggyBac*-genrated CAR19-T cell therapy in RR DLBCL, and the additive effects of lenalidomide maintenance.

### Patient Perspective

When I failed to prepare for ASCT, and then suffered the second relapse and didn’t respond to the following chemotherapy, I felt so helpless. The outcome of patients with RR DLBCL is dismal, especially for patients with MYC, BCL2 and BCL6 rearrangements and TP53 mutuation. I am glad to participate in the study and has received the *piggyBac*-generated CAR19-T cell therapy. Although I attained CR in the 2nd month, I demanded maintenance treatment in view of my experience of two relapses. My doctors gave me oral lenalidomide in the 4th month, but I discontinued after one course due to some side effects. I still keep CR for over 2 years and I hope that my case will give some inspiration for physicians and patients all over the world.

## Data Availability Statement

The original contributions presented in the study are included in the article/supplementary material. Further inquiries can be directed to the corresponding authors.

## Ethics Statement

The studies involving human participants were reviewed and approved by the Ethics Committee of the Union Hospital affiliated to Huazhong University of Science and Technology, Wuhan, China. The patients/participants provided their written informed consent to participate in this study. Written informed consent was obtained from the individual(s) for the publication of any potentially identifiable images or data included in this article.

## Author Contributions

YH and HM conceived and designed the study. CL and YS performed data analysis and wrote the paper. JWa contributed imaging interpretation. LT and HJ performed data collection. TG, LL, YW, and LX participated in patient management. LA analyzed the flow results. JWa, ZL, and QQ provided the CAR19-T cell products. All authors contributed to the article and approved the submitted version.

## Funding

This work was supported by grants from Key Special Project of “Research on Prevention and Control of Major Chronic Non-infectious Diseases” (No. 2019YFC1316203 to HM, No. 2019YFC1316204 to YH).

## Conflict of Interest

Authors YS, JW, ZL, and QQ are employed by Shanghai Cell Therapy Group Co., Ltd.

The remaining authors declare that the research was conducted in the absence of any commercial or financial relationships that could be construed as a potential conflict of interest.

## Abbreviations

ABC, activated B-cell; ASCT, autologous stem cell transplantation; CAR19, CD19-directed chimeric antigen receptor; CR, complete remission/response; CT, computed tomography; DLBCL, diffuse large B cell lymphoma; DHL, double-hit lymphoma; R-CHOP, rituximab, cyclophosphamide, adriamycin and prednisone; R-DHAP, rituximab, dexamethasone, cytarabine and cisplatine; R-ICE, rituximab, ifosfamide, carboplatine and etoposide; FISH, Fluorescence *in situ* hybridization; IHC, immunohistochemistry; ORR, overall response rate; OS, overall survival; PET, positron emission tomography; RR, relapsed/refractory; SCM, stem-cell memory; THL, triple-hit lymphoma.
